# Biochemical Aspects of the Soybean Response to Herbivory Injury by the Brown Stink Bug *Euschistus heros* (Hemiptera: Pentatomidae)

**DOI:** 10.1371/journal.pone.0109735

**Published:** 2014-10-21

**Authors:** Renata Velozo Timbó, Marcelo Hermes-Lima, Luciano Paulino Silva, Angela Mehta, Maria Carolina Blassioli Moraes, Débora Pires Paula

**Affiliations:** 1 Department of Cell Biology, University of Brasília, Campus Universitário Darcy Ribeiro, Brasília, Distrito Federal, Brazil; 2 Department of Biological Control, Embrapa Genetic Resources and Biotechnology, Brasília, Distrito Federal, Brazil; United States Department of Agriculture, Beltsville Agricultural Research Center, United States of America

## Abstract

Plant defense response is an elaborate biochemical process shown to depend on the plant genetic background and on the biological stressor. This work evaluated the soybean biochemical foliar response to brown stink bug herbivory injury through an analysis of redox metabolism and proteomic 2DE profiles of susceptible (BRS Silvania RR) and resistant (IAC-100) varieties. The activity of lipoxygenase-3, guaiacol peroxidase, catalase and ascorbate peroxidase was monitored every 24 h up to 96 h. In the susceptible variety, injury caused an increase in the activities of lipoxygenase 3 and guaiacol peroxidase, no change in ascorbate peroxidase, and a decrease in catalase. In the resistant variety, injury did not cause an alteration of any of these enzymes. The proteomic profiles were evaluated after 24 h of injury and revealed to have a similar proportion (4–5%) of differential protein expression in both varieties. The differential proteins, identified by mass spectrometry, in the susceptible variety were related to general stress responses, to plant defense, and to fungal infections. However, in the resistant variety, the identified change in protein profile was related to Calvin cycle enzymes. While the susceptible variety showed adaptive changes in redox metabolism and expression of stress-responsive proteins, the resistant showed a defense response to circumvent the biological stressor.

## Introduction

Stink bugs (Hemiptera: Pentatomidae) are one of the most important pests of soybean in Brazil [1]. Pentatomids feed on various structures of the host plant, mainly fruits and immature seeds, affecting their quality, development and maturation [2]. The brown stink bug *Euschistus heros* is considered one of the main soybean pests [3], [4]. Information about the soybean defense mechanisms to this herbivore when it colonizes in the late vegetative stages is important to support pest management.

Several varieties of soybean have been studied regarding their defense response to herbivores in order to identify herbivory resistant varieties [5], [6], [7], [8]. These studies have mostly focused on primary and secondary metabolite production as parts of the plant defense strategy aiming to support environmentally friendly soybean production. Plant defense to herbivory injury involves two major systems: (i) the direct defense, involving structural components (e.g. thorns, trichomes), and production of primary metabolites (e.g. proteinase inhibitors, antioxidant enzymes) and non-volatile secondary metabolites (e.g. phenolic acids and isoflavonoids) [9], [10]; and (ii) the indirect defense, which is related to the production of volatile organic compounds (VOCs), such as (*E*,*E*)-α-farnesene, methyl salicylate and *cis*-jasmone, which attract natural enemies (predators and parasitoids) of the herbivores that feed on the plants, or in some cases act as herbivore repellents [6], [11].

Plant defense response varies according to the plant genetic background, and the type of stressor [10]. Therefore, it is valuable to study the response elicited by the key herbivores in different varieties. Biochemical studies on plant defense have focused on evaluating the activity of enzymes related to the redox metabolism (including antioxidant enzymes) [9], [12]–[14], such as lipoxygenase (LOX), peroxidase (POD), glutathione reductase (GR), ascorbate peroxidase (APX), and catalase (CAT), as well as on the modification of protein profiles [15]. However, despite the significant economic impact of the damage caused by stink bugs to soybean [16], [17], there are no reports regarding the soybean redox response and the regulation of protein expression induced by stink bug herbivory.

Plants differ in resistance to their key herbivorous pests according to tolerance, non-preference and antibiosis [18]. In this work we used two soybean varieties with large differences in antibiosis resistance to brown stinkbug: resistant IAC-100 and susceptible BRS Silvania RR. IAC-100 was developed by crossing the parental varieties IAC-12 and IAC 78-2318, showing resistance to chewing and sucking insects [19]. BRS Silvania RR was developed for glyphosate tolerance [20]. The goal was to characterize the biochemical effects of the brown stink bug herbivory injury on the foliar redox response, through the analysis of the activity of lipoxygenase-3 (LOX3), guaiacol peroxidase (GOPx), catalase (CAT) and ascorbate peroxidase (APX), as well as on the foliar protein expression, through two-dimensional electrophoresis and mass spectrometry.

## Materials and Methods

### Soybean cultivation and stink bug rearing

The seeds of IAC-100 and BRS Silvania RR were germinated in a neutral pH germination paper. Seedlings with equivalent development were transplanted to plastic pots of 350 mL containing mixture of soil and organic substrate (in a proportion of 3∶1), maintained in a germination chamber at 25±2°C and 13 h photoperiod, and irrigated every two days. The bioassays were conducted with plants in vegetative stage 3 (V3, first three trifoliolate leaves fully expanded). Stink bugs colonize soybeans during the vegetative stage, during which soybean plants produce higher amount of volatiles compared to the reproductive stage, and are known to have their indirect defense elicited by injury of the brown stink bug [10], [17], [21]. Understanding the plant defense mechanisms during this stage could help control stink bug pest populations that result in the reproductive stage.

The brown stink bugs were obtained from a laboratory colony at Embrapa Genetic Resources and Biotechnology (Brasília, Brazil), reared according to Moraes *et al*. [18] in plastic cages (26×22 cm) containing cotton fiber soaked in water and fed on peanut seeds [*Arachis hypogaea* (L.)], soybean grains [*Glycine max* (L.) Merrill.], sunflower seeds [*Helianthus annus* (L.)] and green beans [*Phaseolus vulgaris* (L.)], at 26±2°C, 60±10% RH and 14 h photoperiod.

### Herbivory bioassays

The herbivory bioassays consisted of no injury (control) or injury to individual V3 plants of the two soybean varieties by three male adult brown stink bugs. Plants were exposed to stink bugs for 24, 48, 72, or 96 h after which the plants were harvested. Simultaneously, plants were harvested from the control. There were six replicate plants per harvest per treatment in a completely randomized design. Only males were used to prevent possible interference of oviposition on the plants, which could trigger other plant responses [6], [21]. The stink bugs were deprived of food for 24 h before the bioassays. The plants were covered with transparent plastic bags (with micro holes for aeration) to guarantee the constant presence of the stink bugs during the bioassay. All assays were performed in separate germination chambers (FANEM) for each treatment, so that the VOC released could not interfere with the other treatments. All leaves of the six plants per treatment were collected and transferred to 50 mL tubes and immediately frozen in liquid nitrogen and stored at −80°C for further biochemical analysis.

### Quantification of enzyme activities

The enzymatic activity was measured for LOX3, GOPx, APX and CAT in the soybean foliar extracts of three plants from each treatment in the four 24 h sampling intervals. Foliar homogenates were prepared in a mortar containing liquid nitrogen, where the leaves were macerated until the formation of a fine powder. For every 100 mg of macerated leaf, 400 µL of 50 mM potassium phosphate buffer (KPi) containing 10 mM phenylmethanesulfonyl fluoride (PMSF), 5 mM ethylenediaminetetraacetic acid (EDTA) was added. After homogenization, the samples were centrifuged at 14,000×*g* for 15 min at 4°C. The supernatant was quantified by the Bradford method [22] and used in triplicate for each enzyme assay.

The conditions for the enzymatic activity assays are detailed in Table 1. The assays for GOPx, APX and CAT were performed in quartz cuvettes (10×10 mm) by adding 900 µL of KPi pH 7.0 and 1 to 20 µL of foliar supernatant. For LOX3, 2,975 µL Kpi pH 6.5 and 1 to 20 µL of foliar supernatant were used. The reactions were initiated by adding the substrate for each enzyme (100 µL for GOPx, APX and CAT and 25 µL for LOX3) and the absorbance was measured for 150 s at intervals of 10 s with a Smart Plus spectrophotometer (BioRad).

**Table 1:** Enzymatic activity assay conditions in leaves of the soybean varieties BRS Silvania RR and IAC-100 in the V3 stage, with and without (control group) brown stink bug herbivory injury.

Enzyme	Substrate	ε (mM^−1^cm^−1^)	Wavelength (nm)	Final Volume (µL)	References
LOX3	80 mM sodium linoleate	22.00	234	3,000	[23]
GOPx	10 mM hydrogen peroxide +20 mM guaiacol	26.60	470	1,000	[24]
APX	10 mM hydrogen peroxide +40 mM ascorbic acid	14.50	265	1,000	[25]
CAT	10 mM hydrogen peroxide	0.04	240	1,000	[26]

The enzymatic activities (µmol.min^−1^.mg^−1^) were compared by one-way ANOVA, and the paired comparisons among the treatments were performed by Tukey's HSD (*p*<0.05).

### Total protein extraction for the proteomic analysis

The total foliar protein profiles of all treatments (three plants/each) were analyzed after 24 h of bioassay by 2-DE. Total protein extraction was performed adapting the TCA/acetone methodology [27]. According to Wildgruber *et al*. [28], this method inhibits the degradation of proteins by minimizing protease activity (due to low pH), and also improves cell lysis and subsequent precipitation of proteins at −20°C. The leaves from the same treatment were pooled and macerated in liquid nitrogen until the formation of a fine powder and chilled cold acetone solution, containing 10% (w/v) TCA and 0.07% (v/v) β-mercaptoethanol, was added in a proportion of 1∶2 v/v. After vortex homogenization for 5 min and incubation for 24 h at −20°C, the samples were centrifuged at 10,000×*g* for 20 min at 4°C. The pellets were washed three times in cold acetone containing 0.07% (v/v) β-mercaptoethanol and centrifuged at 10,000×*g* for 10 min at 4°C. To each 12 mg of pellets 1 mL of solubilization buffer [8.5 M urea, 2.5 M thiourea, 2% (w/v) CHAPS, 1% (w/v) DTT, 1% (w/v) Serdolit, 2% (v/v) IPG buffer nonlinear 3–11, 1% (w/v) bromophenol blue] [29] was added and incubated at room temperature for 1 h under gentle stirring. After centrifugation at 14,000×*g* for 1 h at 25°C, the supernatants were collected and the proteins quantified by the Bradford method [22].

### Two-dimensional electrophoresis (2-DE) and gel image analysis

The isoelectric focusing (IEF) was carried out in 13 cm Immobiline DryStrips pH 3–11 NL (GE Healthcare). The strips were hydrated overnight with 250 µL of solubilization buffer containing 500 µg of total protein extract (three strips for each treatment). The IEF was conducted at 20°C using the Multiphor II Electrophoresis System (GE Healthcare) coupled to a cooling system bath in one step of 300 V for 1 min and one step of 3,500 V for 5.5 h (all at 2 mA and 5 W). After IEF, the strips were reduced by the addition of 8 mL of equilibration buffer [50 mM Tris-HCl pH 8.8, 6 M urea, 30% (v/v) glycerol, 2% (w/v) SDS] containing 1% (w/v) DTT under gentle agitation for 15 min at room temperature; and alkylated by adding 8 mL of equilibration buffer containing 2.5% (w/v) iodoacetamide under gentle agitation for 15 min. The equilibrated strips were transferred to a 12.5% denaturaing polyacrylamide gel and electrophoresis was performed at 25°C in Tris/glycine/SDS buffer on a multiple Ettan DALTsix (GE Healthcare), coupled to a cooling bath system, according to manufacturer's recommendation. For molecular mass reference, the BenchMark Protein ladder (Invitrogen) was applied on a side of the gel using filter paper. The electrophoresis was carried out in two steps: first at 10 mA/80 V for 1 h and 40 mA/500 V for 4.3–6 h. The 2-DE gels were stained for two days in a solution of 0.8% (w/v) Coomassie Brilliant Blue G-250, 20% (v/v) ethanol, 1.6% (v/v) phosphoric acid, 8% (w/v) ammonium sulfate, and rinsed five times in water to remove excess dye.

The 2-DE gel images were obtained by ImageScanner III (GE Healthcare). Spot detection and measurements, as well as spot matching in the three gel images per treatment, were performed using the ImageMaster 2D Platinum 7.0 software (GE Healthcare). A spot was considered differential when its intensity and area were different in the injured and uninjured gels. The comparison of the total average number of spots between the injured and uninjured treatments was performed by one-away ANOVA (*p*<0.05).

### Protein identification

For the soybean variety BRS Silvania RR the differential spots were excised and sliced into small fragments. Spot destaining was conducted by successive washes in 30% ethanol and incubation in 50% (v/v) acetonitrile solution containing 25 mM ammonium bicarbonate under agitation for 15 min. The gel fragments were dehydrated with 100% acetonitrile for 10 min under agitation and dried in a centrifugal evaporator for 20 min. The spots were rehydrated with 50 µL of hydrolysis solution consisting of 50 mM Tris-HCl pH 7.6 added to 1 mM calcium chloride and 0.5 µL of trypsin at 1 µg/µL (Sequencing Grade Modified Trypsin, Promega), which was previously heated to 30°C for 15 min, and to 50 µL of 50 mM ammonium bicarbonate. The samples were incubated at 37°C for 14 to 18 h. For the soybean variety IAC-100, trypsin hydrolysis was carried out using the Trypsin Profile IGD Kit (Sigma), according to the manufacturer instructions.

The trypsin-hydrolyzed peptides were analyzed by MALDI-TOF mass spectrometry using an UltraFlex III MALDI TOF/TOF (Bruker Daltonics) controlled by the software FlexControl 3.3.108.0 using the following parameters: mass range 600-3,500 Da; RP_PEPMIX method; 200 automatic shooting at a frequency of 200 Hz, laser intensity between 20–45%; deflection mass of 600 Da; reflective mode; and positive external calibration by peptide calibration standard I. The method to obtain the MS/MS data was the LIFT; AUTO_LIFT method; using the following parameters: 2,000 shots for which, 400 and 1,600 shots for the precursor and the fragments, respectively; laser intensity between 22–55%; and the other parameters were the same as mentioned above. The peaks list files containing the m/z ratios of precursor ions and MS/MS fragmented ions were used to search similar oligopeptides in the National Center for Biotechnology Information (NCBI) database sequences using BLASTp and Mascot software. The parameters used in the Mascot program for MS/MS were: (i) NCBI-nr database, (ii) Viridiplantae taxonomy, (iii) fixed modifications: carbamidomethylation of cysteine, (iv) variable modification: oxidation of methionine, (v) monoisotopic mass spectra data, (vi) mass tolerance of 150 ppm, (vii) fragment tolerance of ±0.05 Da. Only significant hits, as defined by Mascot (*p*<0.05), were accepted.

## Results and Discussion

### Redox response

**Figure 1:**
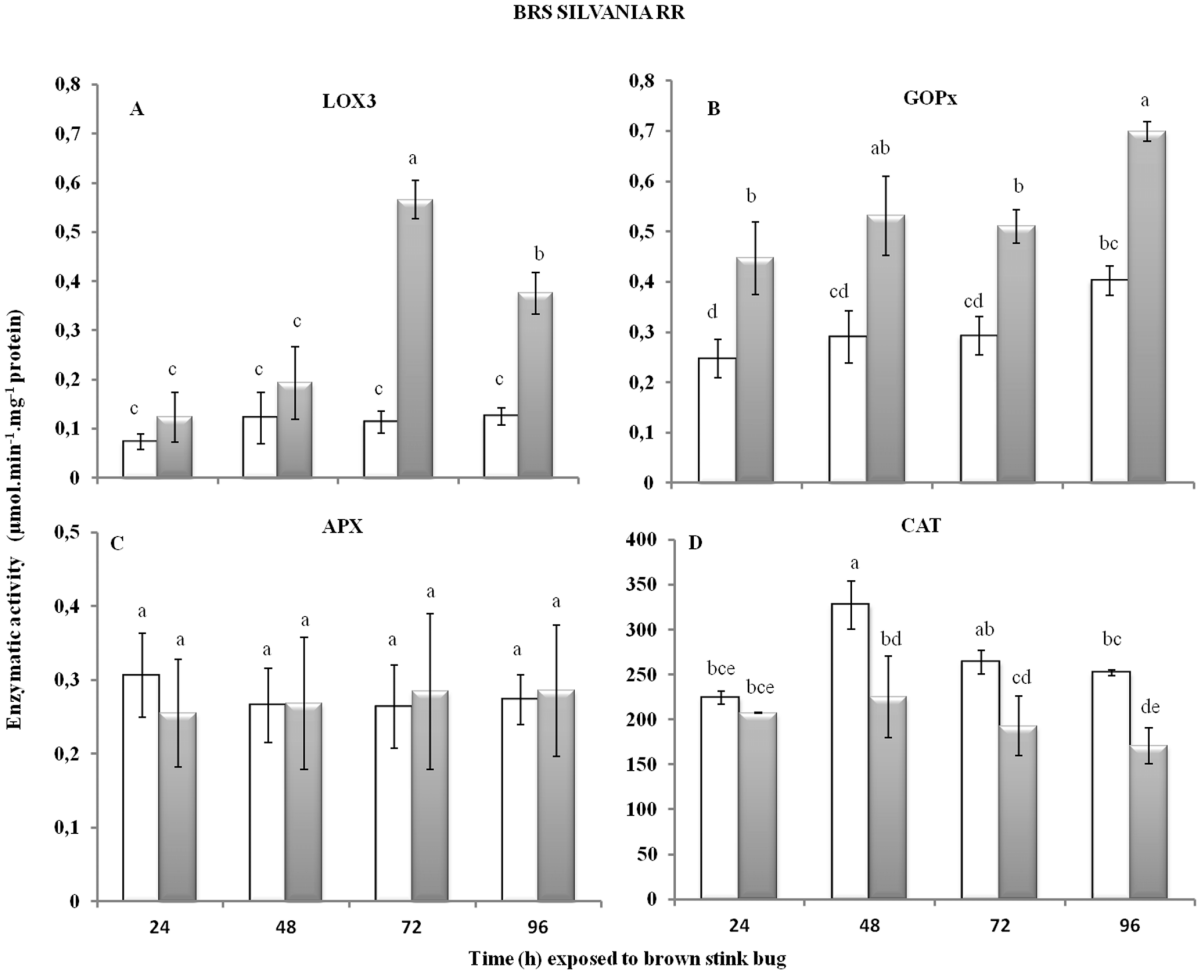
Enzymatic activity for (A) LOX3 (F_7,16_ = 2.32; *p* = 0.0779), (B) GOPx (F_7,16_ = 1.01; *p* = 0.4629), (C) APX (F_7,16_ = 3.35; *p* = 0.0213), and (D) CAT (F_7,16_ = 1.78; *p* = 0.1609) in leaves of BRS Silvania RR soybean variety (V3) with or without brown stink bug herbivory injury along different sampling periods. Injured is represented in grey and not injured in white. Bars with the same letter are not significantly different by Tukey's HSD (*p*<0.05).

**Figure 2:**
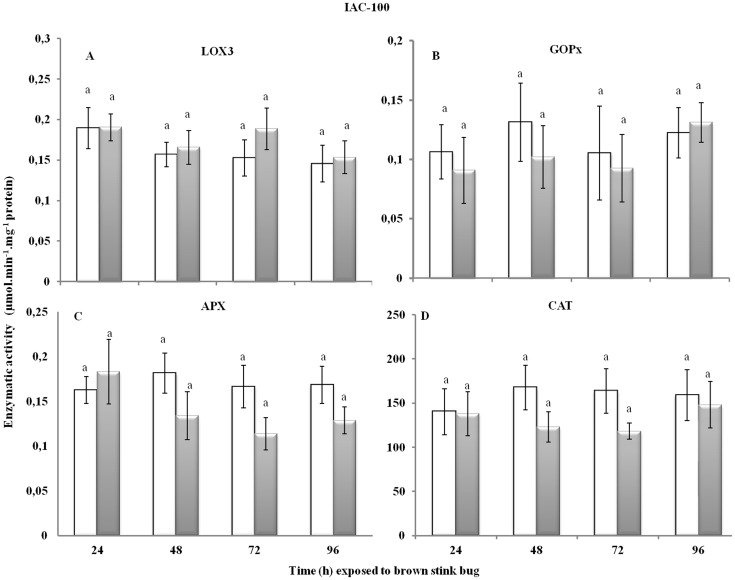
Enzymatic activity for (A) LOX3 (F_7,16_ = 34.52; *p*<0.0001), (B) GOPx (F_7,16_ = 29.6; *p*<0.0001), (C) APX (F_7,16_ = 0.16; *p* = 0.9907), and (D) CAT (F_7,16_ = 12.59; *p*<0.0001) in leaves of IAC-100 soybean variety (V3) with and without brown stink bug herbivory injury along different sampling periods. Injured is represented in grey and not injured in white. Bars with the same letter are not significantly different by Tukey's HSD (*p*<0.05).

The herbivory injury by the stink bug affected the foliar enzymatic response (*p*<0.05) in the susceptible BRS Silvania RR variety (Figure 1). The LOX3 activity was significantly higher at 72 h of herbivory, with a decline at 96 h, but still significantly higher than in the first 48 h of injury and in the uninjured control. The GOPx activity did not differ over the time of injury, but was higher along the time evaluated compared to the uninjured plants. On the other hand, APX activity was not affected during the herbivory period, and CAT activity decreased after 48 h of injury compared to uninjured plants. In contrast, the herbivory injury by the brown stink bug did not affect the foliar activity of any of the enzymes evaluated even after 96 h of injury (Fig. 2) for the resistant IAC-100 variety, suggesting that the redox metabolism was not affected in this variety under *E. heros* feeding injury.

From the enzymes studied, LOX3 and GOPx have a clear relation to the plant defense response and an enhanced activity under herbivory injury has been reported [30]–[32]. LOX3 catalyzes the production of jasmonic acid (JA) from linolenic acid, which stimulates the expression of genes responsible for producing defense compounds and is related to plant defense against herbivorous insects, and GOPx activates defense genes [30]–[32]. Chen *et al*. [24] observed that herbivory by *Melanocallis caryaefoliae* (Hemiptera: Aphididae) induced higher activity of LOX3 and GOPx in susceptible pecans, although in moderate and high resistance pecans the GOPx activity did not change. Similarly, Ni *et al*. [33] reported increased activity of GOPx, CAT and polyphenol oxidase in wheat leaves after herbivory by *Diuraphis noxia* (Hemiptera: Aphididae), and that peroxidase activity in barley leaves was significantly higher in susceptible than in resistant plants after herbivory by the same aphid species.

Although uninjured BRS Silvania RR generally had higher activity of three enzymes related to the redox metabolism than IAC-100, LOX3 activity was higher in uninjured IAC-100 than in BRS Silvania RR. This result may be directly related to resistance, because one of the functions of the lipoxygenase pathways the production of JA. The constitutive high activity of lipoxygenase in plants is related to their resistance against insects [34], which may help to explain the resistance of IAC-100 against stink bugs [6], [16] and why it produces high level of induced plant volatiles when injured by them, which were not detected in BRS Silvania RR [6].

APX catalyzes the redox reaction between hydrogen peroxide and ascorbate [35]. Bi & Felton [36] reported that APX activity in soybean leaves increased over time after herbivory by *Helicoverpa zea* (Lepidoptera: Noctuidae), with maximum activity at 96 h. In our results, the activity of APX was not affected by stink bug herbivory on the susceptible soybean variety. Stink bugs are piercing-sucking insect, therefore provoking less damage to plants compared to chewing insects [37], and possibly inducing lower levels of hydrogen peroxide in the injury process.

CAT performs two major functions: (i) decomposition of hydrogen peroxide (a product of photorespiration and other plant metabolic activities) and (ii) oxidation of hydrogen donors such as methanol, ethanol, formic acid, phenols, with consumption of hydrogen peroxide [25], [38]. An increase in CAT activity may be related to the control of hydrogen peroxide levels (which can be stressful at high concentrations) formed in response to herbivory injury [39]. In herbivory studies on various plant species, CAT activity remained unchanged [12], [24], [40], increased [33] or decreased [36], [37]. In our study, a decrease in CAT activity was observed in response to stink bug injury only in the susceptible variety, showing that the varieties are managing the intracellular hydrogen peroxide levels differently. The observed increase in GOPx activity could be compensating the reduction in CAT levels in BRS Silvania RR leaves.

Overall, the susceptible soybean variety (BRS Silvania RR) showed redox gene expression changes, while the resistant variety (IAC-100) did not. The fluctuation in the activity of the redox metabolism enzymes might be associated with redox stress, which could be an indication of herbivory susceptibility. In the resistant variety, the redox genes might be constitutively expressed, so the stink bug injury might not produce enough reactive oxygen species (ROS) to elicit changed expression of these genes. Additional assessment of the redox alterations generated by herbivory across time would include measures such as protein carbonyls, lipid peroxidation and oxidized glutathione (GSSG), which are also classic indicators of redox oxidative stress, as well as other antioxidant enzymes, such as superoxide dismutases and peroxyredoxins [26].

### Expression of foliar proteins

In both varieties, the foliar protein profile changed after 24 h of herbivore injury by the brown stink bug. Not all differentially expressed proteins could be identified, even when using the soybean genome reference database, possibly due to their low concentration or even absence in the database.

**Figure 3:**
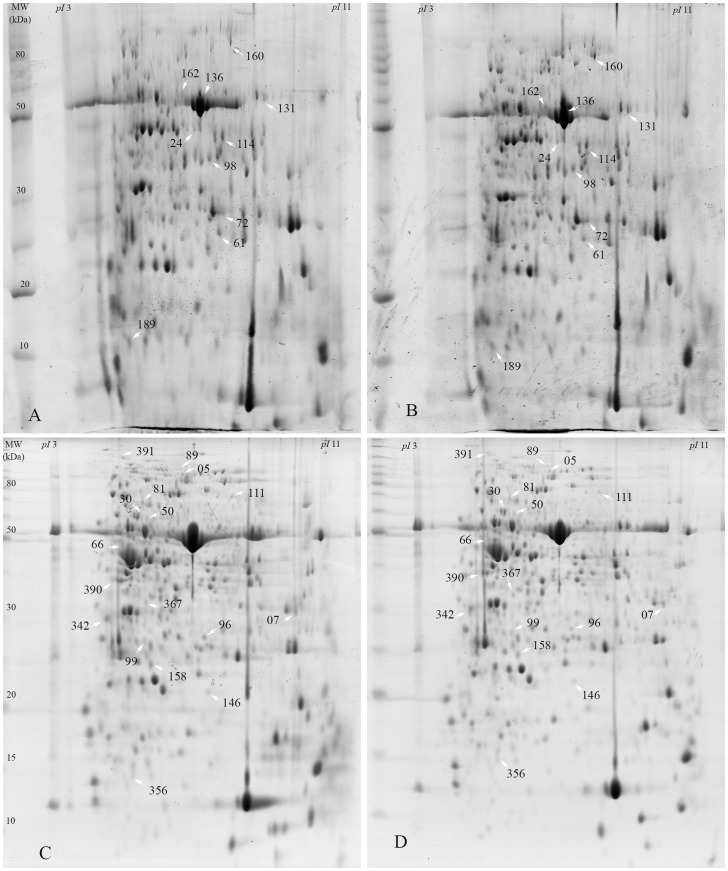
Foliar protein profiles (2-DE, 500 µg) of soybean varieties (V3). Variety IAC-100: (A) injured and (B) not injured, from the total average of 191 (±2) protein spots, 10 were differentially expressed and two identified (Table 2). Variety BRS Silvania RR: (C) injured and (D) not injured, from the total average of 386 (±4) protein spots, 17 were differentially expressed and 11 identified (Table 3). The differentially expressed proteins are indicated with white arrows and numbers, which correspond to identification numbers listed in the respective table.

The 2-DE gel analyses of the BRS Silvania RR foliar protein profiles had an average of 386 protein spots per gel, while IAC-100 showed an average of 191 protein spots per gel, and most proteins were observed in the pH range from 4 to 8 and in the molecular mass range from 15 to 100 kDa (Fig. 3). The gel replicates of uninjured and injured plants had a high reproducibility: *r* ^2^ = 0.95 and 0.96, respectively for the susceptible; and *r* ^2^ = 0.93 and 0.97, respectively for the resistant.

When the foliar protein profile of the injured susceptible plants was compared to the uninjured profile, 17 proteins were differentially expressed, of which eight were up-regulated (Table 2). The identified up-regulated proteins were the chaperonin HSP20, glycine-rich 2b-like protein, both related to biotic and abiotic stress responses [41], [42], and one hypothetical protein (gi|147800453).

**Table 2:** Identification of differentially expressed proteins by MS/MS in BRS Silvania RR soybean leaves (V3) (*p*<0.05).

Spot number	Protein identification	Accession (NCBI)	Molecular mass (kDa) theoretical/experimental	p*I* theoretical/experimental	Peptide sequence	Mascot/Blast Score	Search Type	RMS Error (ppm)	E-value	Expression level (%)
07	GATase1-like domain	gi|255639997	27.0/31.5	5.43/9	LSDFFEILATSEDR	117	MS/MS	44.40	4e-09	↓ 16.7
35*	Unknown	gi|40538952	-/57.8	-/5.6	GFIMKGATNATVDSMV	-	MS/MS	-	6e-13	↑ 30.4
66	CP4-EPSPS	gi|18266432	47.6/47.7	5.13/5	SAVLLAGLNTPGITTVIEPIMTR	57	MS/MS	12.80	2.2e-3	↓ 54.6
81*	70 kDa chaperonin-like	gi|473930207	-/64.6	-/5.6	AVITVPAYFNDAQR	-	MS/MS		1e-06	↓ 56.0
99*	STI-Soybean trypsin inhibitor	gi|351726331	19.8/26.8	-/5.5	GFPITISSPAR	-	MS/MS	-	0.003	↓ 24.0
111*	Unknown	gi|242054087	-/74.6	-/7.7	LSGIIPVHIGKLTK	-	MS/MS	-	5e-06	↓ 15.5
146	Glycine-rich protein 2b-like	gi|356538622	19.3/21.6	6.29/7.09	SLAEGESVEFAIESESDGR	132	MS/MS	11.60	1.6e-10	↑ 250.0
158	20 kDa chaperonin-like	gi|356556406	26.6/21.6	7.79/5.76	DKPSIGTVIAVGPGHLDEEGNR	146	MS/MS	0.86	3.2e-12	↑ 88.0
342	Unknown	gi|1478004532	50.0/31.0	5.43/4.7	SLLYVETADRPGLLVDLVK	90	MS/MS	4.51	1.6e-06	↓ 68.1
356	Glycine-rich RNA-binding	gi|351725469	15.8/15.0	6.58/5.3	AFSQYGEIVETK	77	MS/MS	20.90	2.9e-05	↓ 35.0
367	SGNH plant lipase-like	gi|255646252	40.0/33.4	5.83/5.6	AFFVFGDSLVDNGNNNFLATTAR	85	MS/MS	50.90	4.2e-06	↑ 58.9

* Identification by similarity search with BLASTp using peptide sequence obtained by manual *de novo* sequencing.

The identified down-regulated proteins were CP4-EPSPS enzyme, a protease inhibitor, related to plant defense to herbivory, a hypothetical hydrolase function, related to plant defense against fungal infections [43], a heat-shock chaperonin 70 kDa protein (HSP), a glycine-rich RNA binding protein (GRP), related to general plant stress responses, a type 1 glutamine amidotransferase-like (GATase1) and three unknown hypothetical proteins (gi|40538952, gi|242054087, gi|147800453).

CP4-EPSPS participates in the shikimic acid pathway synthetizing the aromatic amino acids phenylalanine, tyrosine and tryptophan, which act as precursors for a wide range of products such as alkaloids, the plant hormone auxin, phytoalexins [44], flavonoids, and phenolic compounds such as lignin [45], and secondary volatile compounds such as methyl salicylate and indole, which are involved in indirect plant defense [6], [20], [21]. Michereff *et al.* [6] showed that the soybean variety BRS Silvania RR did not release methyl salicylate or indole when injured by the brown stink bug. Therefore the decrease of CP4-EPSPS in BRS Silvania RR might be related to its susceptibility to stink bugs [6], [16]. HSPs are not exclusively related to heat stress, and some are induced by stresses such as injury, drought, and salinity [46]. In plants, GRPs are responsible for regulating cellular processes of genic regulation post-transcription, and translation [42], [47], as well as cell elongation [48], protoxylem development [49], [50], signal transduction [51], RNA chaperone activity [52], plant defense against pathogens [53], RNA binding activity [54], among others. Sachetto-Martins *et al*. [55] reported that the expression of GRP is regulated by a number of external stimuli, including stress by cold, water, high salinity, injury, and viral infection. The GATase1 belongs to a large group of enzymes capable of removing ammonia from glutamine and to transfer this group to other substrates to form amino acids, purine, pyrimidine nucleotides, and coenzymes [56]. Further studies are necessary to understand why these proteins were increased or decreased when the plants suffer injury by brown stink bug.

When the protein profiles of the injured and uninjured resistant plants were compared, 10 proteins had quantitative differential expression levels, of which four were increased in the injured plants. Two differentially expressed proteins were identified as ribulose bisphosphate carboxylase oxygenase (RuBisCO), which increased, and glyceraldehyde-3-phosphate dehydrogenase (GAPDH), which decreased (Table 3).

**Table 3:** Identification of differentially expressed proteins (by MS/MS) in IAC-100 soybean leaves (V3) (*p*<0.05).

Spot number	Protein identification	Accession (NCBI')	Molecular mass (kDa) theoretical/experimental	p*I* theoretical/experimental	Peptide sequence	Mascot Score	Search Type	RMS Error (ppm)	E-value	Expression level (%)
114	GAPDH	gi|66026	36.0/42.7	6.66/7.2	K.VIAWYDNEWGYSQR.V	64	MS/MS	61.7	8.4e-4	↓ 32.0
136	RuBisCO large subunit	gi|131898	52.0/53.9	6.23/6.24	K.TFQGPPHGIQVER.D	107	MS/MS	11.0	9.1e-08	↑ 33.0

RuBisCO and GAPDH are enzymes in the Calvin cycle [57]. RuBisCO catalyzes the reaction of one carbon dioxide with ribulose-1,5-bisphosphate to form 3-phosphoglycerate (3-PGA), which in turn is catalyzed by phosphoglycerate kinase to 1,3-diphosphoglycerate. This compound, after catalysis by GAPDH produces glyceraldehyde-3-phosphate [43]. The increased expression of RuBisCO, and decreased expression of GAPDH, increases the level of the intermediate 3-phosphoglycerate. 3-Phosphoglycerate is metabolized during glycolysis into phosphoenolpyruvate, which in turn can be used in the shikimic acid pathway. Similar to what occurred in BRS Silvania RR, a protein involved in the shikimic pathway was affected by a piercing-sucking insect on IAC-100 variety. In general, sucking insects act on the shikimic acid pathway, and chewing insects act on the jasmonic acid pathway. Our results corroborate this hypothesis, because the main proteins identified here are involved in the shikimic acid pathway. However in contrast to BRS Silvania RR, the shikimic pathway was up-regulated in IAC-100. Michereff *et al*. [6] showed that IAC 100 injured by brown stink bugs produced higher amounts of methyl salicylate, and this volatile appears to be involved in the indirect defense of soybean.

Overall, the resistant variety (IAC-100) did not exhibit physiological stress to feeding injury by the brown stink bug, and seemed to shift its metabolism to produce secondary compounds for insect defense. In contrast, the susceptible variety (BRS Silvania RR) exhibited signs of physiological stress, both in its enzymes related to redox metabolism and in the protein profile response, which could affect its subsequent development in long term exposure to herbivory injury.

## Conclusions

This work assessed the foliar redox response and protein expression profile of two soybean varieties (IAC-100, resistant, and BRS Silvania RR, susceptible, at stage V3) under herbivory injury by the brown stink bug. As could be expected from previous reports, the two soybean varieties responded differently at the biochemical level to herbivory by the brown stink bug. The susceptible variety BRS Silvania RR showed adaptive changes in the activity of enzymes related to redox metabolism and in the expression of stress-responsive proteins, probably to minimize biochemical damage caused by herbivory injury. On the other hand, in the resistant variety, the redox genes could be constitutively expressed, and the stink bug injury might not produce enough ROS to elicit changed expression of these genes. Overall, the susceptible variety responded to herbivory in ways that could compromise its development in the long term, while the resistant variety IAC-100 showed a defense response oriented to circumvent the biological stressor. Both varieties were injured by a piercing-sucking insect and the proteins regulated by this stress were linked to the shikimic acid pathway corroborating the hypothesis that sucking insects induce the shikimic acid route of defense more than the jasmonic acid route defense.
